# Supplementary material for: Detection of Bacterial α-l-Fucosidases with an Ortho-Quinone Methide-Based Probe and Mapping of the Probe-Protein Adducts
Source: Molecules. 2022 Feb 28;27(5):1615. doi: 10.3390/molecules27051615 (PMC8911971; doi:10.3390/molecules27051615)
Supplement: Supplementary file 1 [file molecules-27-01615-s001.zip › molecules-1593393-supplementary.pdf]

Supporting information:

Detection of Bacterial α-L-Fucosidases with an *Ortho*-Quinone Methide-Based Probe and Mapping of the Probe-Protein Adducts

Yvette M. C. A. Luijkx [1,†], Anniek J. Henselijn [1,†], Gerlof P. Bosman [1], Dario A. T. Cramer [2], Koen C. A. P. Giesbers [3], Esther M. van 't Veld [4], Geert-Jan Boons [1,5], Albert J. R. Heck [2], Karli R. Reiding [2], Karin Strijbis [3,*] and Tom Wennekes [1,*]

1 Department Chemical Biology and Drug Discovery, Utrecht Institute for Pharmaceutical Sciences and Bijvoet Center for Biomolecular Research, Utrecht University, Universiteitswg 99, 3584 CG, Utrecht, The Netherlands; yvetteluijkx@gmail.com (Y.M.C.A.L.); a.j.henselijn@lic.leidenuniv.nl (A.J.H.); g.p.bosman@uu.nl (G.P.B.); g.j.p.h.boons@uu.nl (G.-J.B.)

2 Department Biomolecular Mass Spectrometry and Proteomics, Bijvoet Center for Biomolecular Research and Utrecht Institute for Pharmaceutical Sciences, Utrecht University and Netherlands Proteomics Center, Padualaan 8, 3584 CH, Utrecht, The Netherlands; d.a.t.cramer@uu.nl (D.A.T.C.); a.j.r.heck@uu.nl (A.J.R.H.); k.r.reiding@uu.nl (K.R.R.)

3 Department Biomolecular Health Sciences, Division Infectious Diseases and Immunology, Faculty of Veterinary Medicine, Utrecht University, Yalelaan 1, 3584 CL, Utrecht, The Netherlands; c.a.p.giesbers@uu.nl

4 Department Biomolecular Health Sciences, Centre for Cell Imaging, Faculty of Veterinary Medicine, Utrecht University, Yalelaan 1, 3584 CL, Utrecht, The Netherlands; e.m.vantveld@uu.nl

5 Complex Carbohydrate Research Center, University of Georgia, Athens, Georgia 30602, GA, The United States.

* Correspondence: k.strijbis@uu.nl (K.S.); t.wennekes@uu.nl (T.W.)

† These authors contributed equally to this work

Table of Content

1. **Section S1.** Supporting figures and schemes
2. **Section S2.** NMR spectra
3. **Section S3.** Protein sequences BfucH and TmFuc
4. **Section S4.** Clustal Omega alignment of BfFucH, TmFuc and AfcA

1. Section S1. Supporting figures and schemes

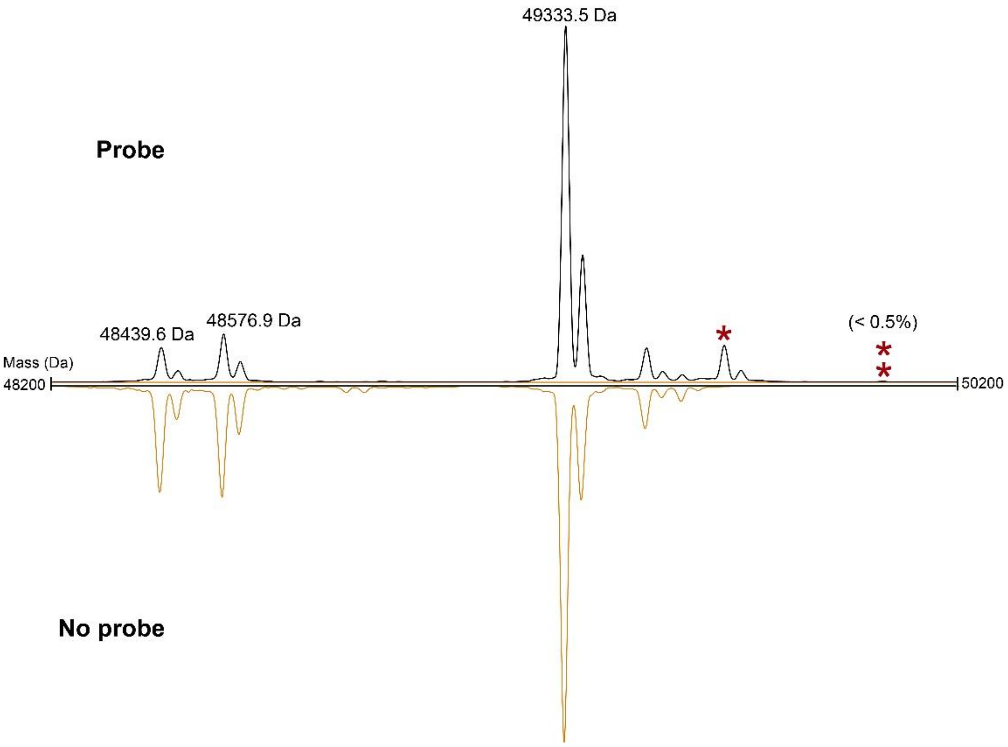

Figure S1. Zero-charge deconvoluted mass spectra of the intact BfFucH analyzed by LC–MS. Shown here are the fucosidase without labelling (top) and after labelling with the **AH062** probe (top). Three variants of the BfFucH were observed, one at a retention time of 9.2–9.6 minutes, representative of the intact molecule (49333.5 Da) and two at a retention time of 9.6–10.1 minutes, representative of a truncated molecule (−756 Da; −893 Da). Red asterisks indicate labeling with the **AH062** probe, corresponding to an approximate mass increase of 350 Da or 700 Da (doubly labeled).

A. W80 – without probe

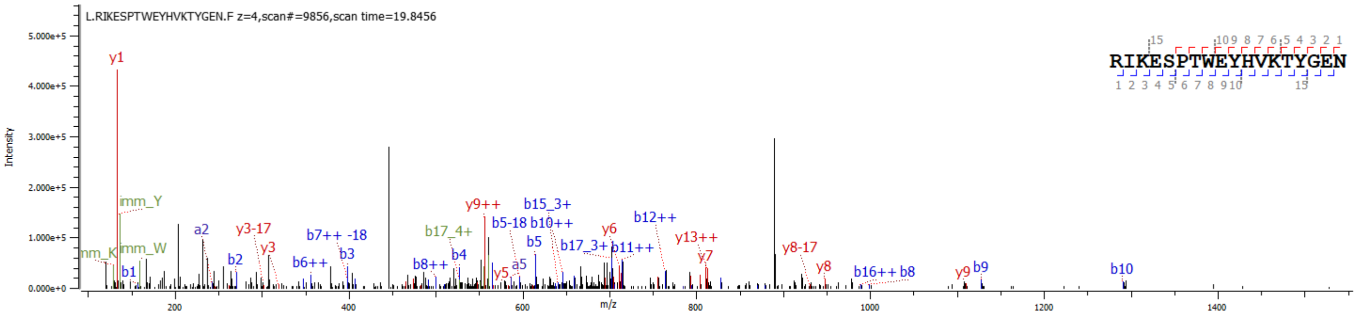

B. W80 – with probe

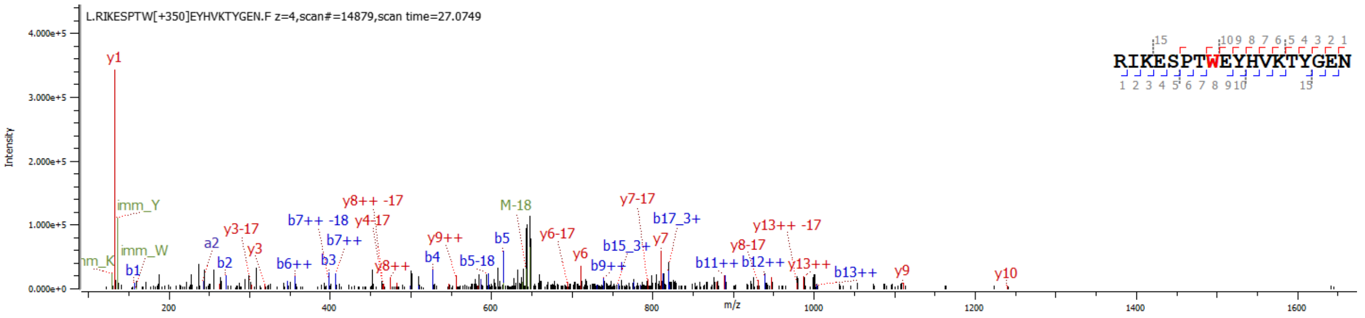

C. W105 – without probe

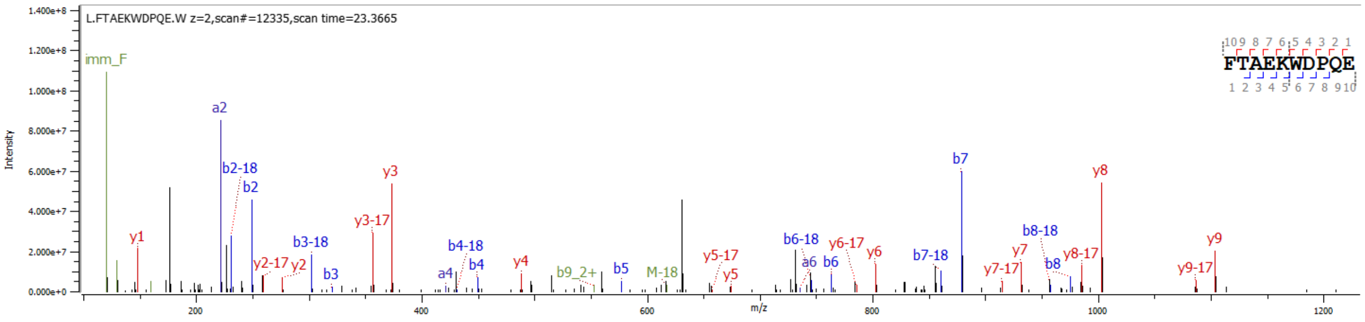

D. W105 – with probe

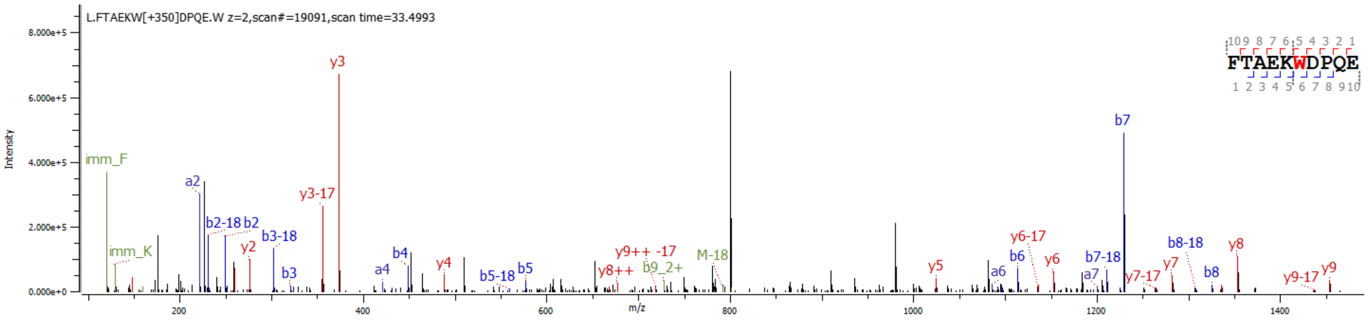

Figure S2 LC MS-MS spectra of pepsin generated peptides from **AH062**-labeled TmFuc indicative for the attachment of the probe to either W80 and W105. Additionally the corresponding non-modified MS-MS spectra are shown. A mass shift corresponding to the mass of the QM probe (350.16 Da) can be observed between the b–and y–ions overlapping W80 and W105.

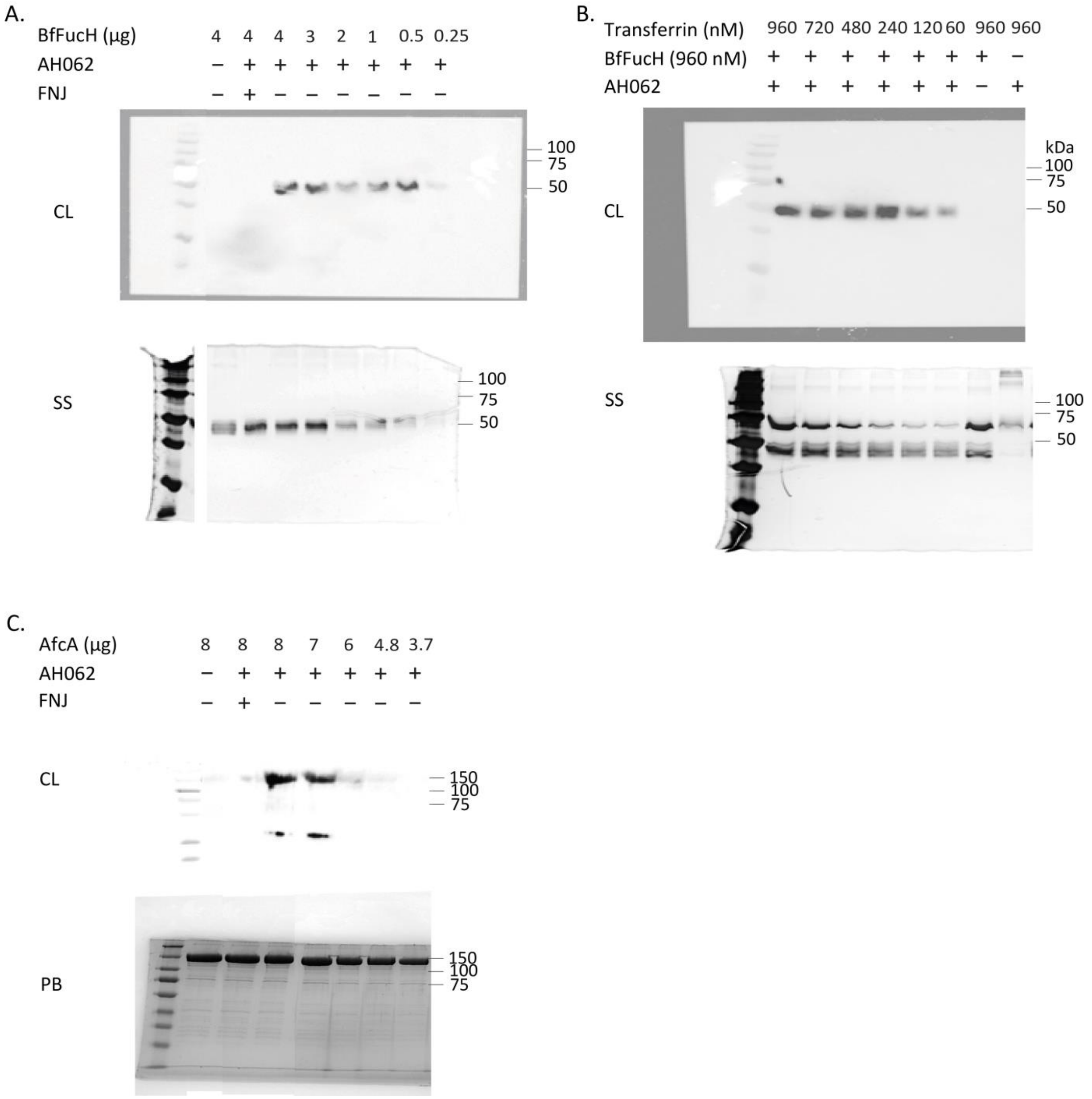

Figure S3. Raw Blots and gels that are presented as cropped images in the main manuscript as Figure 1.

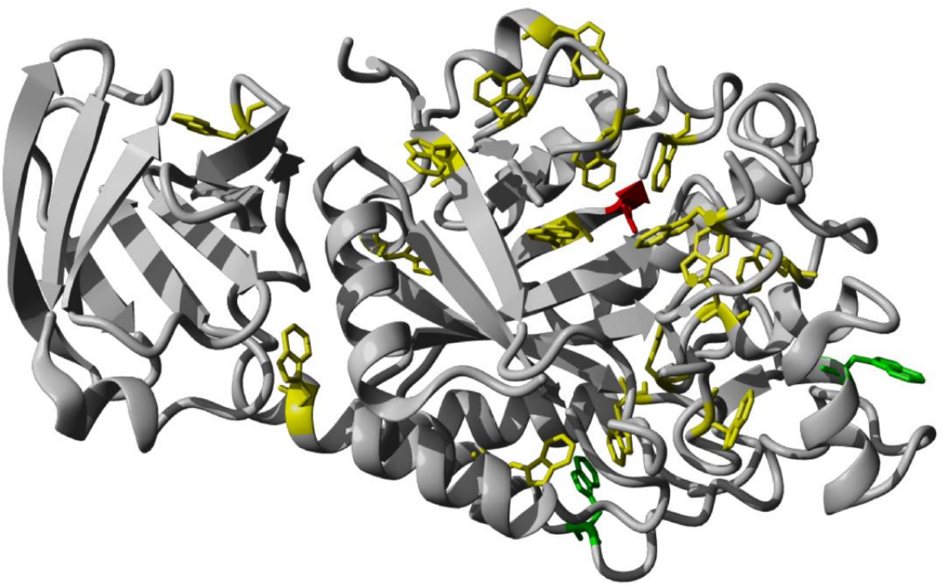

Figure S4. Structure of TmFuc with its active-site catalytic acid D224 annotated in red, residues modified by quinone methide probe AH062 annotated in green and all other Trp residues in yellow. (PDB code 1ODU)

2. Section S2. NMR spectra

^{1}H-NMR spectrum of 5

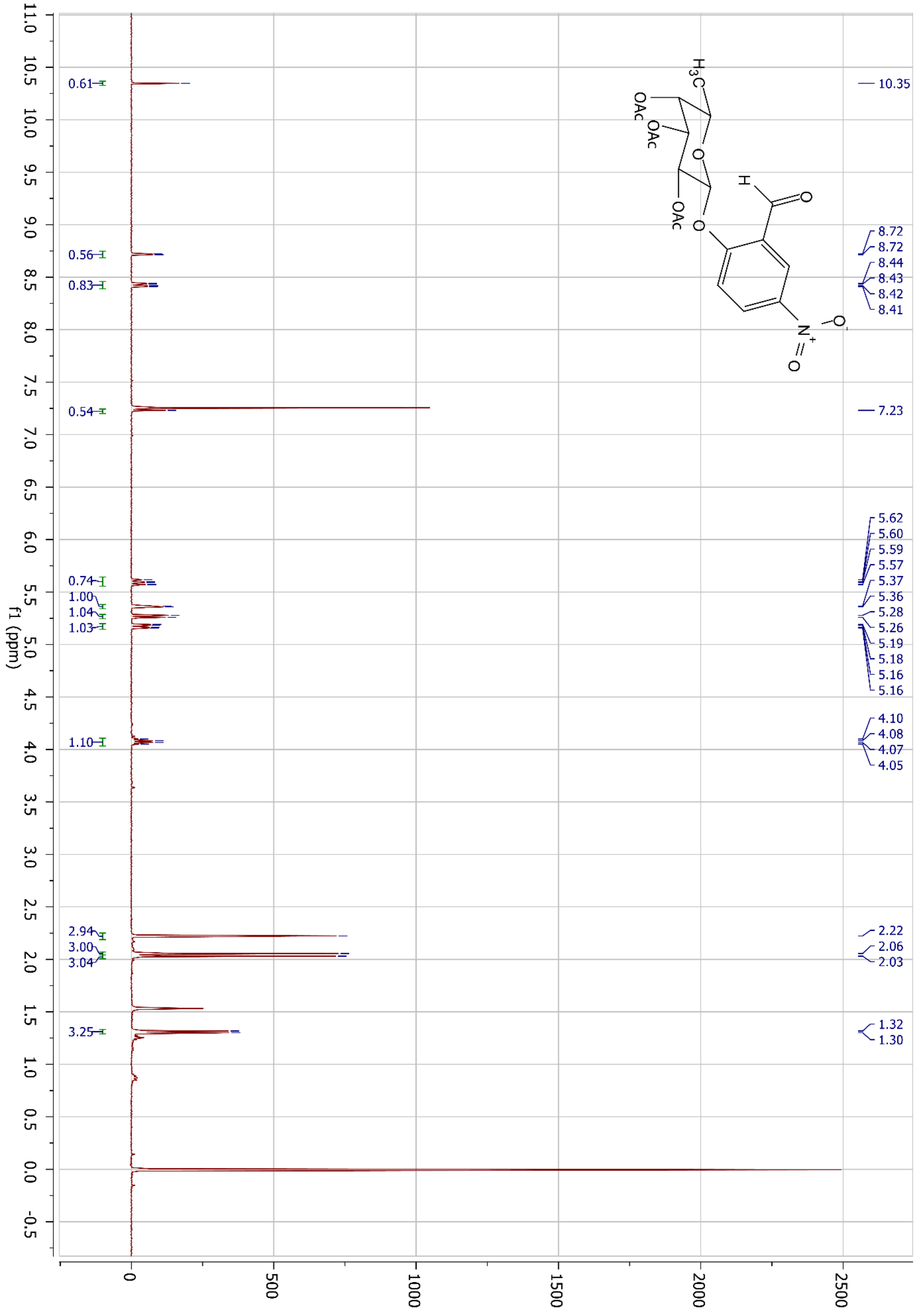

^{1}H-NMR spectrum of 6

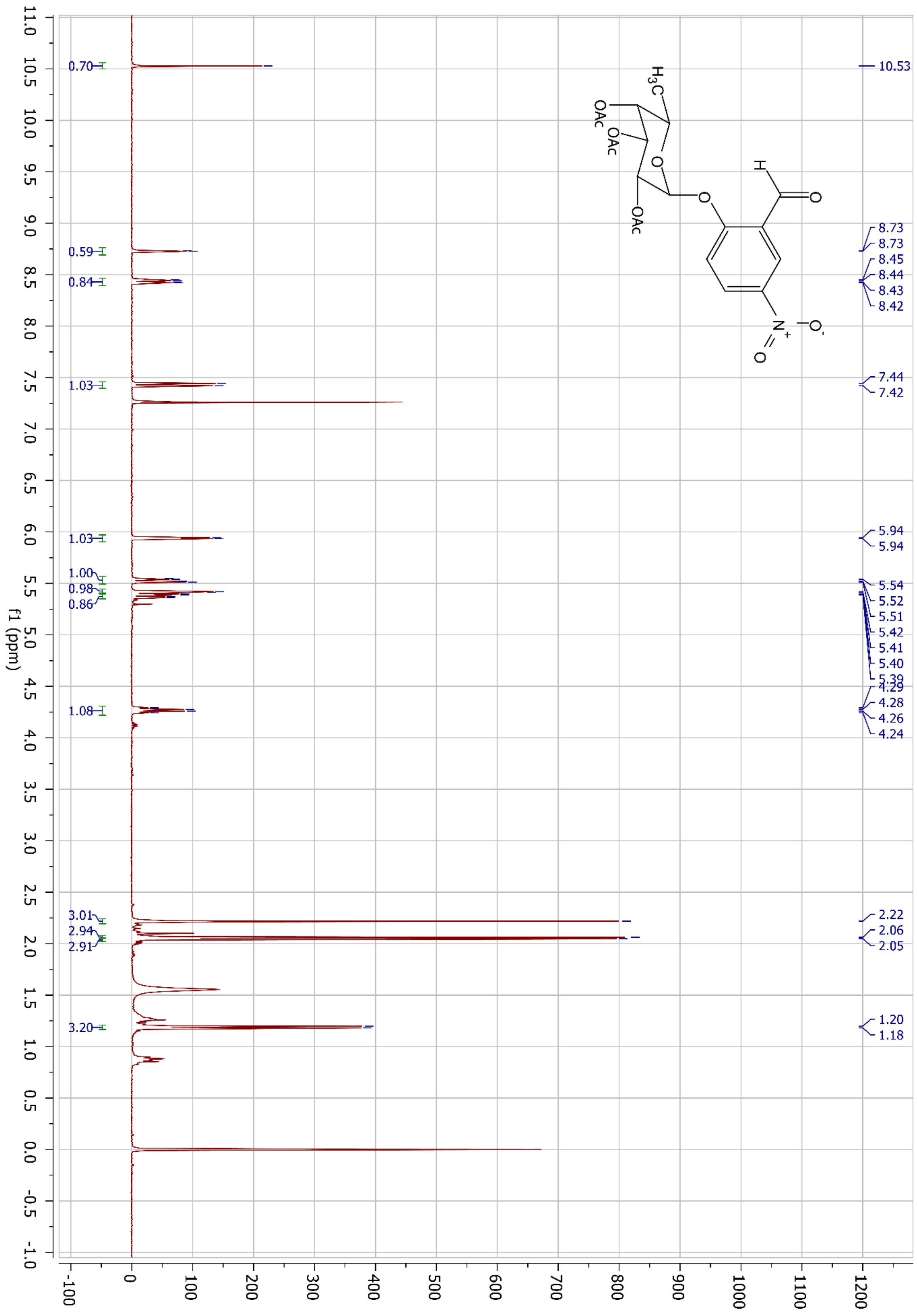

^{1}H-NMR spectrum of 7

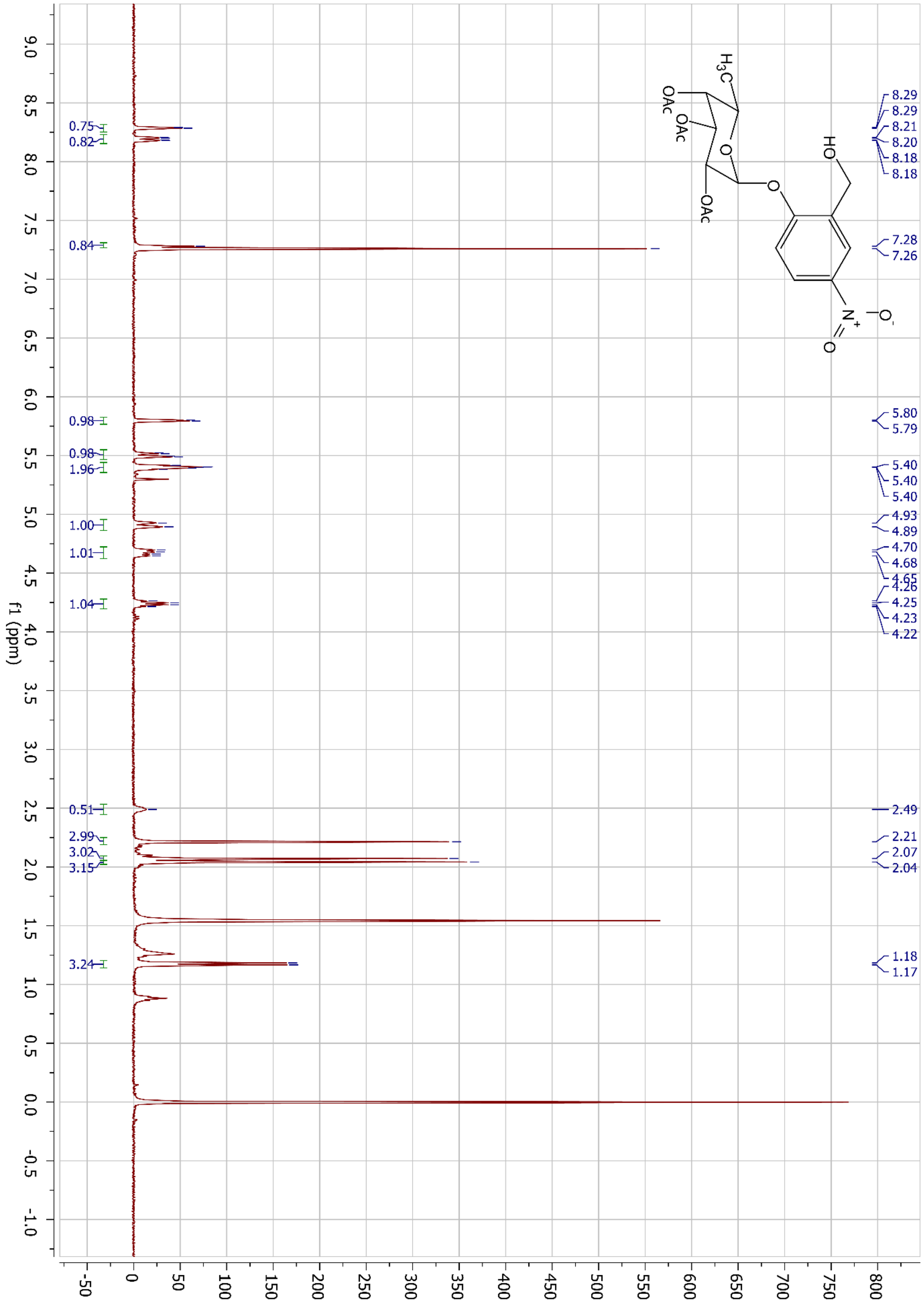

^{1}H-NMR spectrum of 8

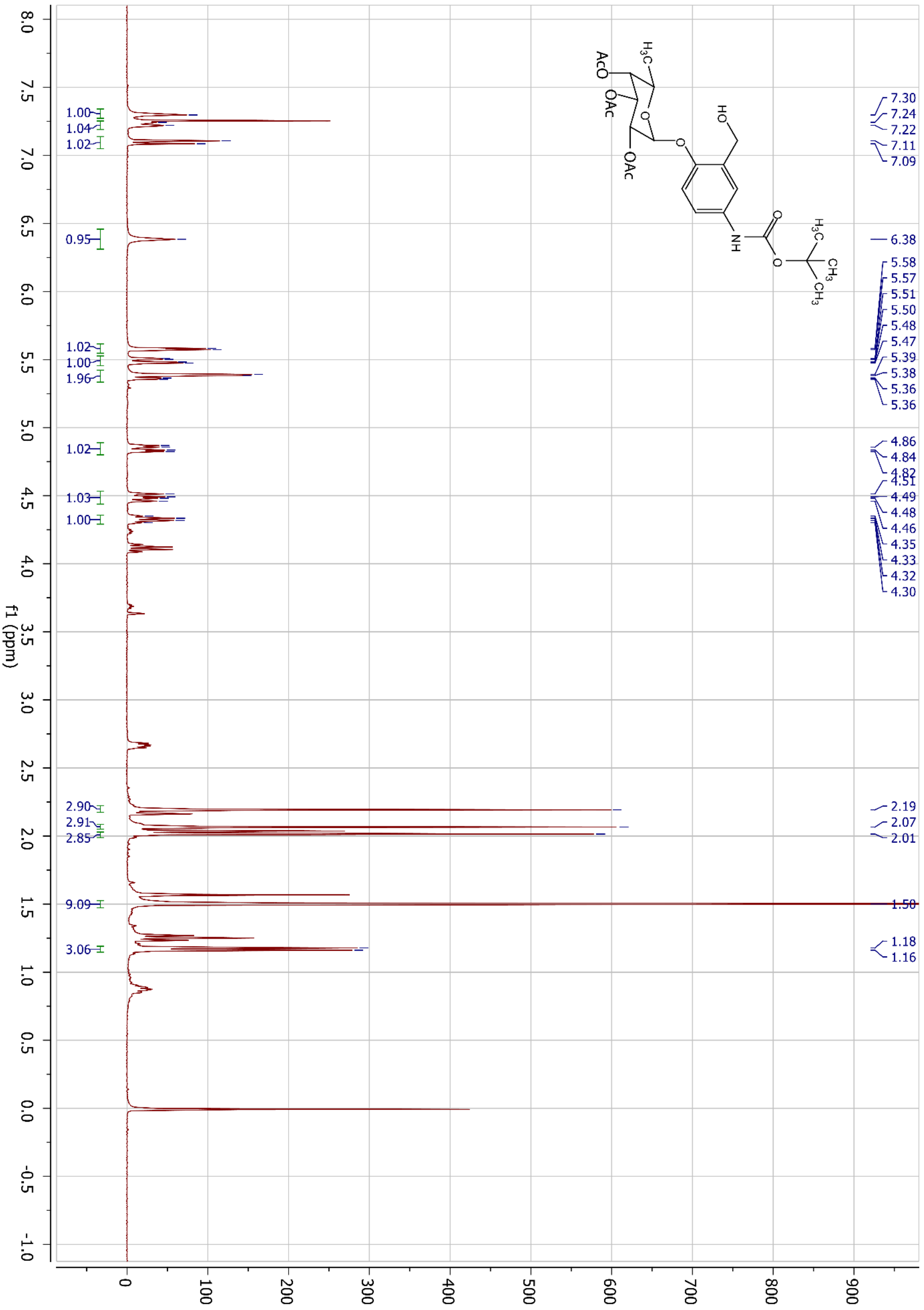

^{1}H-NMR spectrum of 9

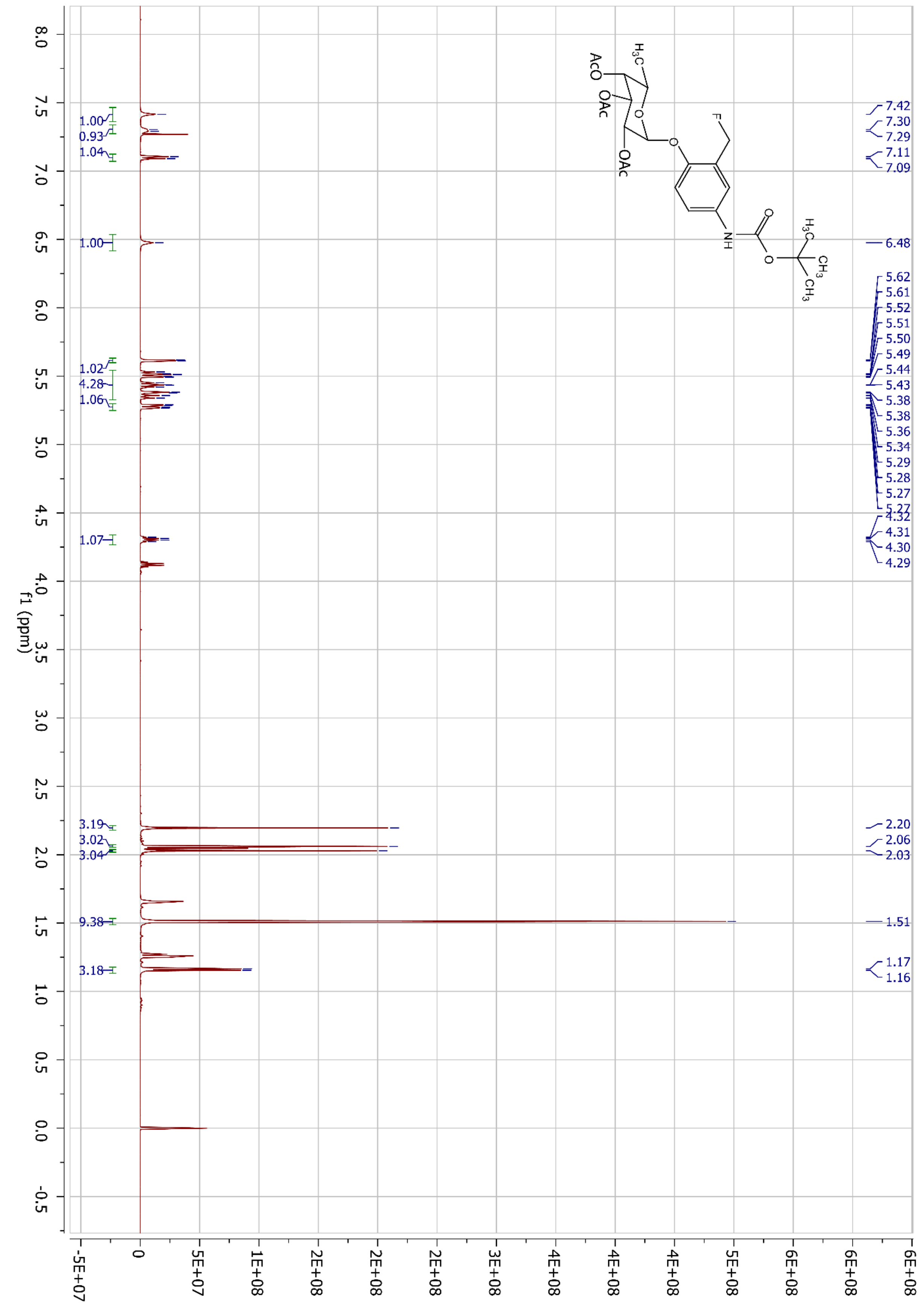

^{1}H and ^{13}C-NMR spectra of 10

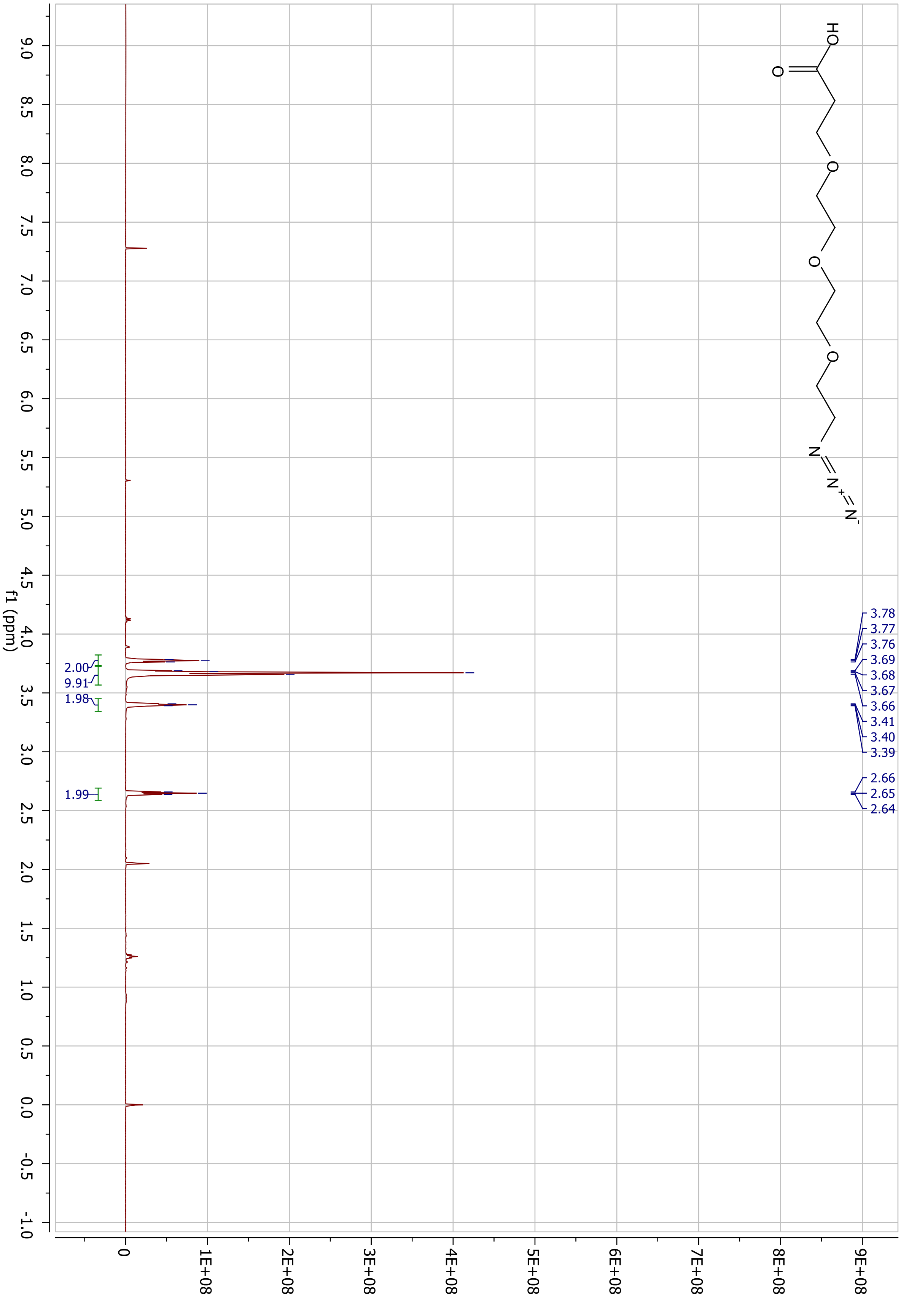

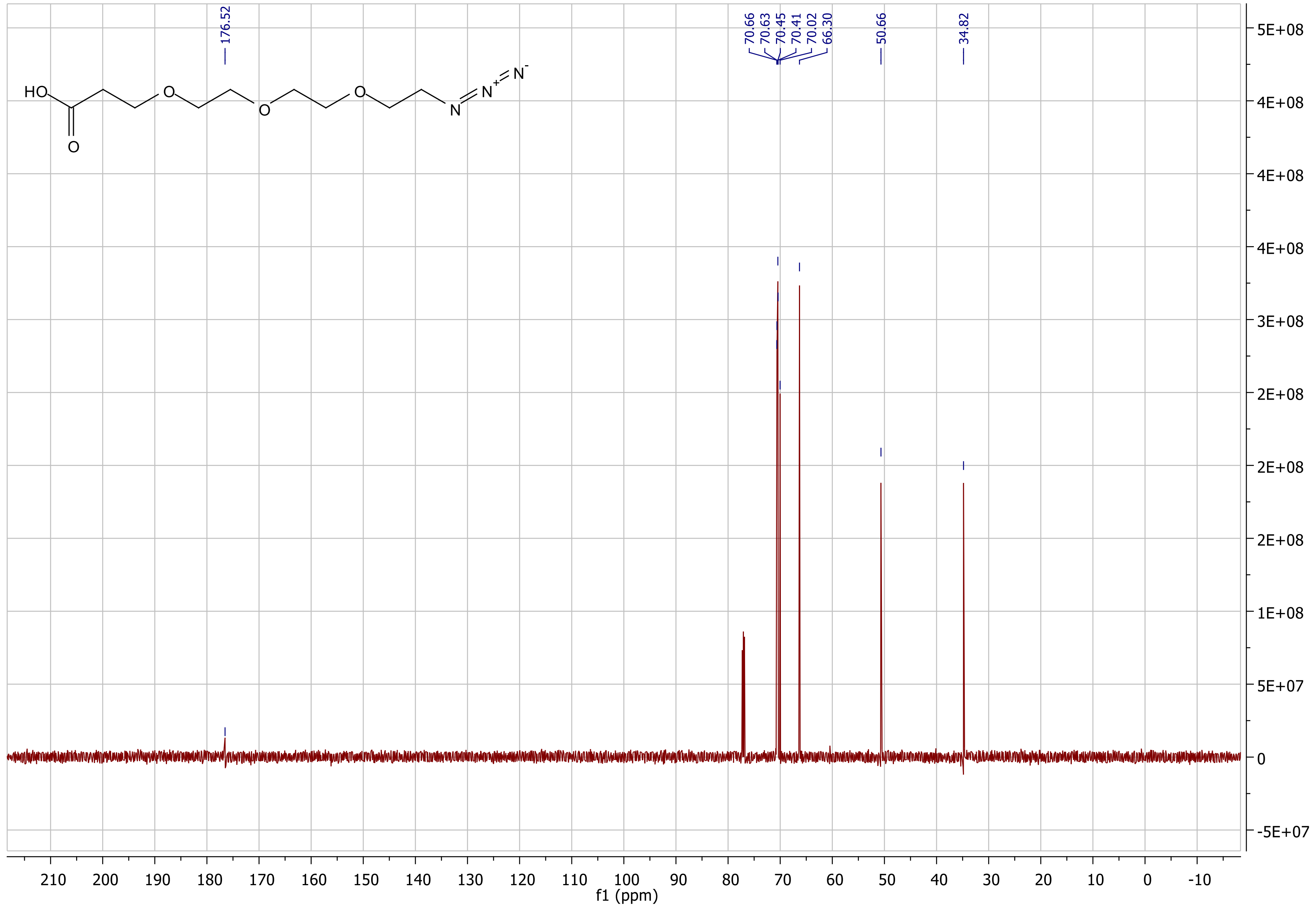

176.52
70.66
70.63
70.45
70.41
70.02
66.30
50.66
34.82
HO
O
O
O
O
N
N
+
N
-
5E+08
4E+08
4E+08
4E+08
3E+08
2E+08
2E+08
2E+08
1E+08
5E+07
0
-5E+07
210
200
190
180
170
160
150
140
130
120
110
100
90
80
70
60
50
40
30
20
10
0
-10
f1 (ppm)

^{1}H, ^{13}C, and ^{19}F-NMR spectra of 11

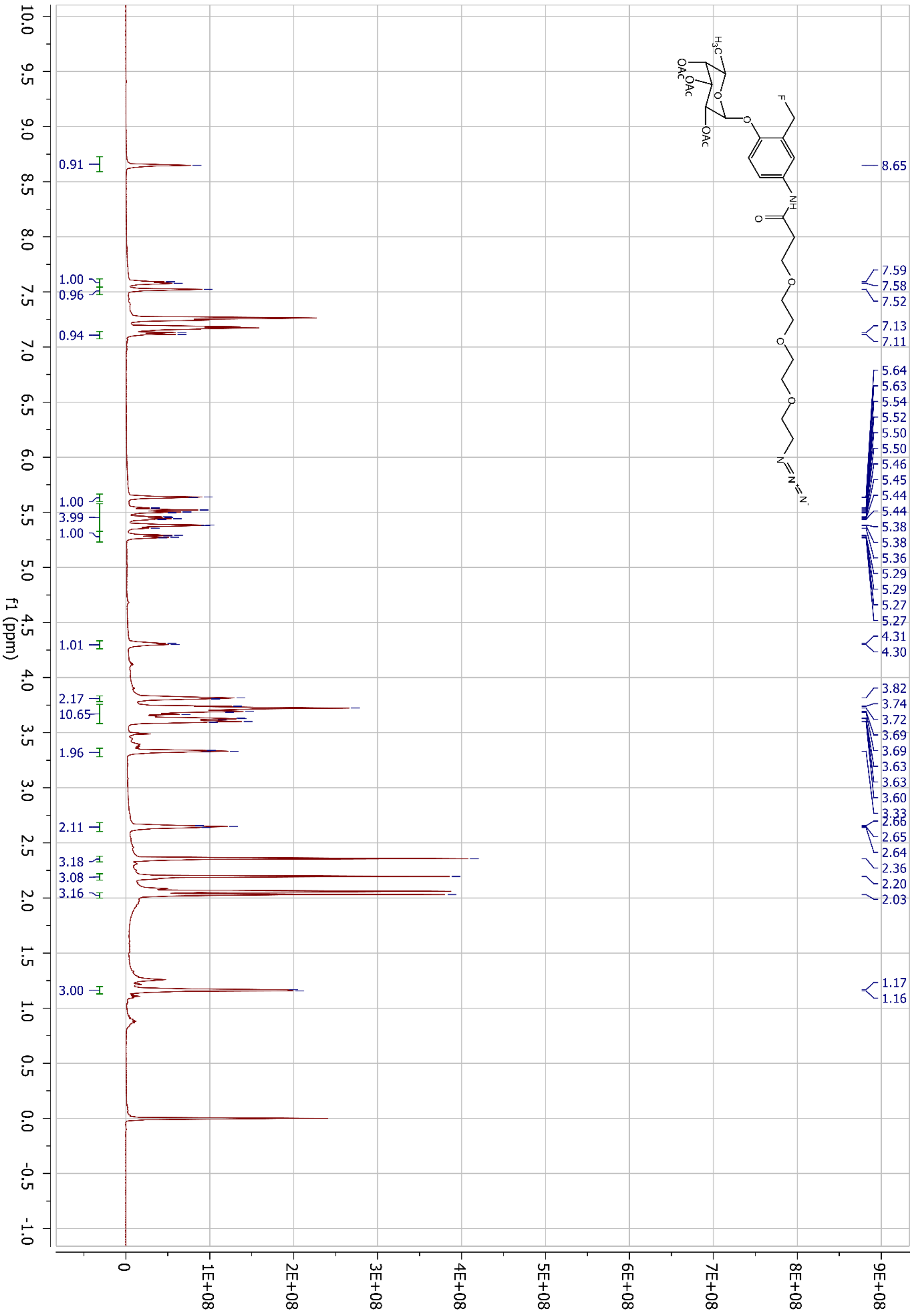

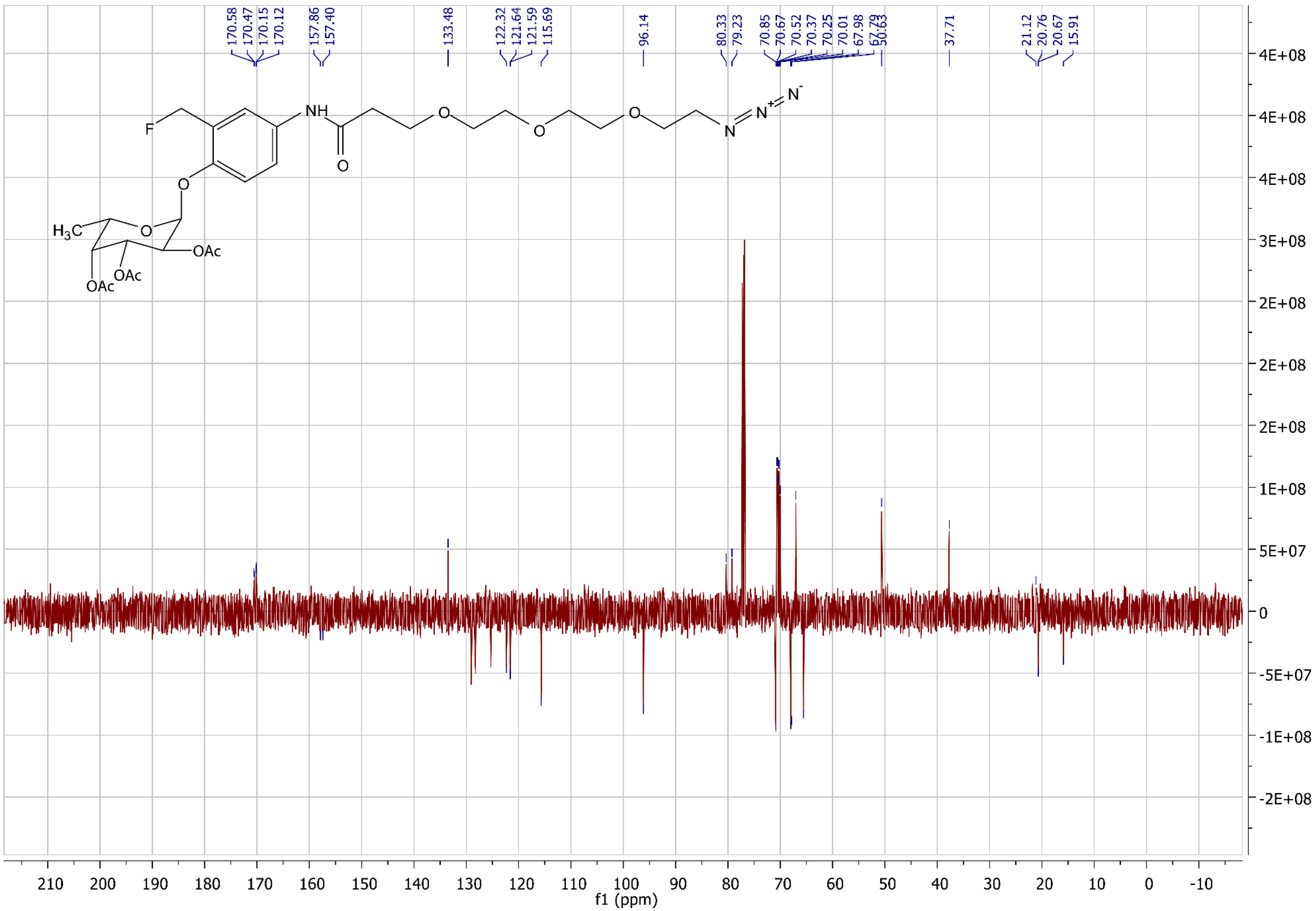
170.58
170.47
170.15
170.12
157.86
157.40
133.48
122.32
121.64
121.59
115.69
96.14
80.33
79.23
70.85
70.67
70.52
70.37
70.25
70.01
67.98
67.79
50.63
37.71
21.12
20.76
20.67
15.91
F
NH
O
N
N^+
N^-
H_3C
OAc
4E+08
3E+08
2E+08
1E+08
5E+07
0
-5E+07
-1E+08
-2E+08
210
200
190
180
170
160
150
140
130
120
110
100
90
80
70
60
50
40
30
20
10
-10
f1 (ppm)

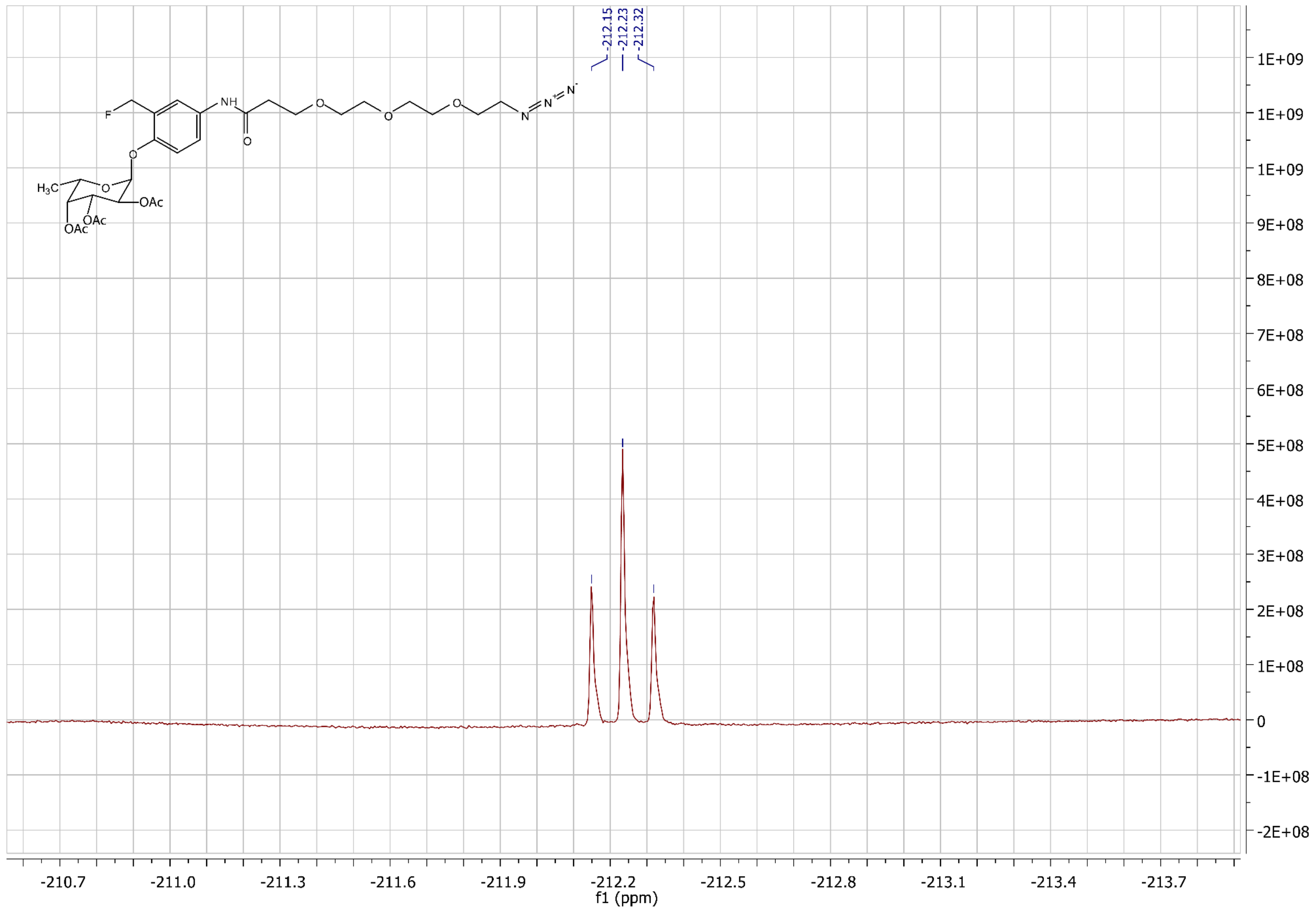
-212.15
-212.23
-212.32
F
NH
O
H3C
OAc
OAc
OAc
N
N
N
-210.7
-211.0
-211.3
-211.6
-211.9
-212.2
-212.5
-212.8
-213.1
-213.4
-213.7
f1 (ppm)
1E+09
1E+09
1E+09
9E+08
8E+08
7E+08
6E+08
5E+08
4E+08
3E+08
2E+08
1E+08
0
-1E+08
-2E+08

^{1}H, ^{13}C, and ^{19}F-NMR spectra of AH062

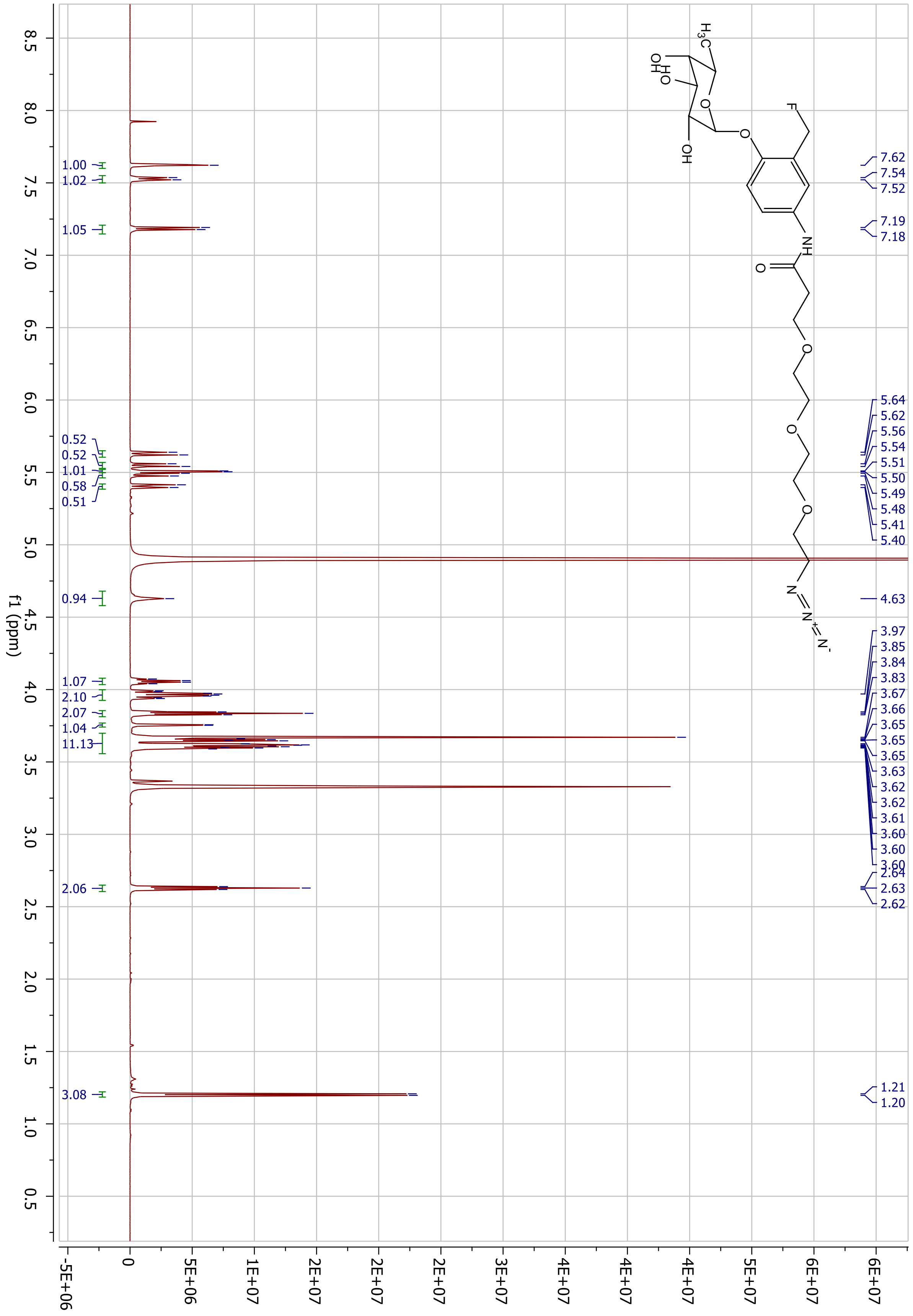

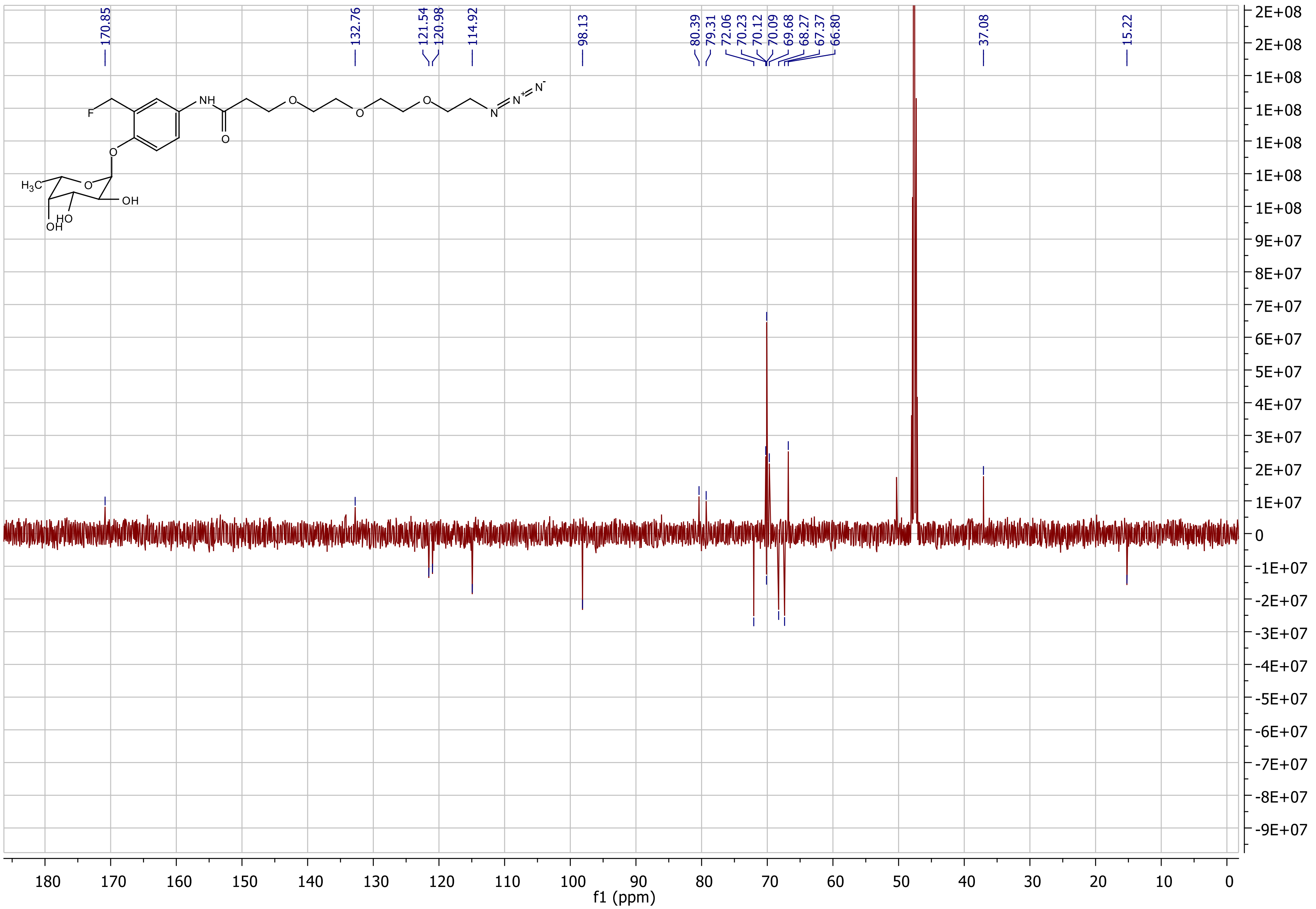
170.85
132.76
121.54
120.98
114.92
98.13
80.39
79.31
72.06
70.23
70.12
70.09
69.68
68.27
67.37
66.80
37.08
15.22
F
NH
O
N
N
N
H3C
OH
HO
OH
f1 (ppm)
180
170
160
150
140
130
120
110
100
90
80
70
60
50
40
30
20
10
0
2E+08
2E+08
1E+08
1E+08
1E+08
1E+08
1E+08
9E+07
8E+07
7E+07
6E+07
5E+07
4E+07
3E+07
2E+07
1E+07
0
-1E+07
-2E+07
-3E+07
-4E+07
-5E+07
-6E+07
-7E+07
-8E+07
-9E+07

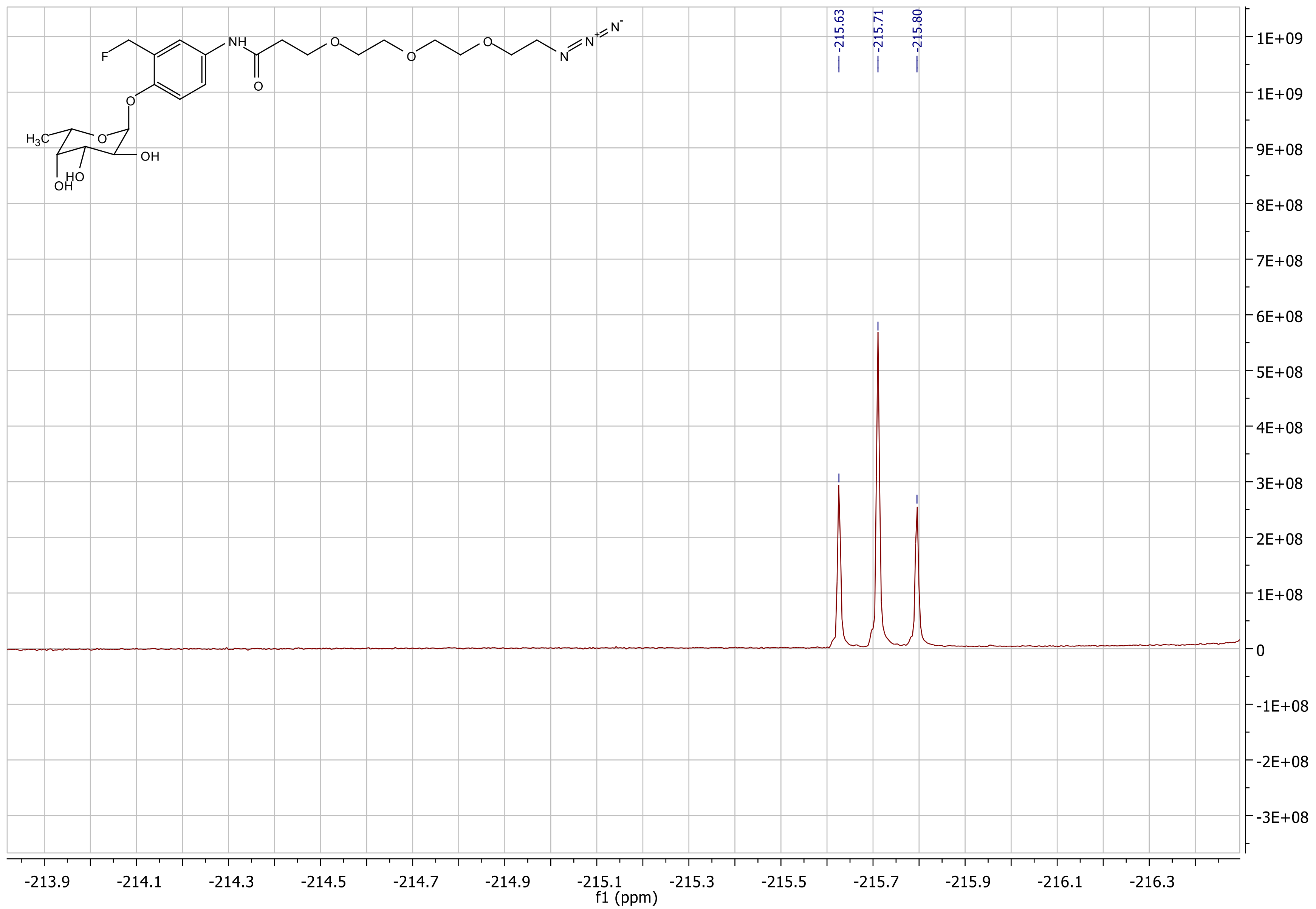
-215.63
-215.71
-215.80
-213.9
-214.1
-214.3
-214.5
-214.7
-214.9
-215.1
-215.3
-215.5
-215.7
-215.9
-216.1
-216.3
f1 (ppm)
1E+09
1E+09
9E+08
8E+08
7E+08
6E+08
5E+08
4E+08
3E+08
2E+08
1E+08
0
-1E+08
-2E+08
-3E+08

3. Section S3. Protein sequences BfFuchH and TmFuc

BfFucH

10 20 30 40 50 60
QQKYQPTEAN LKARSEFQDN KFGIFLH**W**GL YAMLATGE**W**T MTNNNLNYKE YAKLAGGFYP

70 80 90 100 110 120
SKFDADK**W**VA AIKASGAKYI CFTTRHHEGF SMFDTKYSDY NIVKATPFKR DVVKELADAC

130 140 150 160 170 180
AKHGIKLHFY YSHID**W**YRED APQGRTGRRT GRPNPKGDMA HHHHHHSAAL EVLFQG**W**KSY

190 200 210 220 230 240
YQFMNNQLTE LLTNYGPIGA I**W**FDG**WW**DQD INPDFD**W**ELP EQYALIHRLQ PACLVGNNHH

250 260 270 280 290 300
QTPFAGEDIQ IFERDLPGEN TAGLSGQSVS HLPLETCETM NGM**W**GYKITD QNYKSTKTLI

310 320 330 340 350 360
HYLVKAAGKD ANLLMNIGPQ PDGELPEVAV QRLKEVGE**W**M SKYGETIYGT RGGLVAPHD**W**

370 380 390 400 410 420
GVTTQKGNKL YVHILNLQDK ALFLPIVDKK VKKAVVFADK TPVRFTKNKE GIVLELAKVP

430
TDVDYVVELT ID

TmFuc

10 20 30 40 50 60
MISMKPRYKP D**W**ESLREHTV PK**W**FDKAKFG IFIH**W**GIYSV PG**W**ATPTGEL GKVPMDA**W**FF

70 80 90 100 110 120
QNPYAE**W**YEN SLRIKESPT**W** EYHVKTYGEN FEYEKFADLF TAEK**W**DPQE**W**

ADLFKKAGAK

130 140 150 160 170 180
YVIPTTKHHD GFCL**W**GTKYT DFNSVKRGPK RDLVGDLAKA VREAGLRFGV
YYSGGLDWRF

190 200 210 220 230 240
TTEPIRYPED LSYIRPNTYE YADYAYKQVM ELVDLYLPDV L**W**NDMG**W**PEK
GKEDLKYLFA

250 260 270 280 290 300
YYYNKHPEGS VNDR**W**GVPH**W** DFKTAEYHVN YPGDLPGYK**W** EFTRGIGLSF
GYNRNEGPEH

310 320 330 340 350 360
MLSVEQLVYT LVDVVSKGGN LLLNVGPKGD GTIPDLQKER LLGLGE**W**LRK
YGDAIYGTSV

370 380 390 400 410 420
WERCCAKTED GTEIRFTRKC NRIFVIFLGI PTGEKIVIED LNLSAGTVRH FLTGERLSFK

430 440
NVGKNLEITV PKKLLETDSI TLVLEAVEE

4. Section S4. Clustal Omega alignment of BfFucH, TmFuc and AfcA

Highlighted in yellow the tryptophan units in TmarFuc identified in the MS analysis.

```
BbAfcA      MKIEEGKLVIWINGDKGYNGLAEVGKKFEKDTGIKVTVEHPDKLEEKFPQVAATGDGPDI
            60
BfFuc       ------------------------QQKYQ-------------------PTEANLK---AR  14
TmarFuc     --------------------MISMKPRYK-------------------PDWESLREHTVP        21
                          :::            *

BbAfcA      IFWAHDRFGGYAQSGLLAEITPDKAFQDKLYPFTWDAVRYNGKLIAYPIAVEALSLIYNK 120
BfFuc       SEFQDNKFGIFLHWGLYAMLAT------------------------------------GE        38
TmarFuc     KWFDKAKFGIFIHWGIYSVPGWAT---------------PTGELGKVPM---------DA        57
        : . :** : : *: :                          .

BbAfcA      DLLPNPPKTWEEIPALDKELKAKGKSALMFNL--QEPYFTWPLIAADGGYAFKYENGKYD178
BfFuc       WTMTNNN----------------------------LNYKEYAKL-AGGFYPSKFDADKWV           69
TmarFuc     WFFQNPYAEWYENSLR-----IKESPTWEYHVKTYGENFEYEKF-ADLFTAEKWDPQEWA   111
        : *                      :  : *.    *::  ::

BbAfcA      IKDVGVDNAGAKAGLTFLVDLIKNKHMNADTDYSIAEAAFNKGETAMTINGPWAWSNIDT
            238
BfFuc       -------AAIKASGAKYICFTT--RH--------------H--------EG---FSMFDT        95
```

```
TmarFuc     -------DLFKKAGAKYVIPTT--KH-------------H--------DG---FCLWGT  137
              :* .::    :*          :      :*  :.  .*

BbAfcA      SKVNYGVTVLPTFKGQPSKPFVGVLSAGINAASPNKELAKEFLENYLLTDEGLEAVNKDK
           298
BfFuc      KYSDYNI--------VKATPFKRDVVKELADACAKHGI-----KLHFYYSH-IDWYREDA           141
TmarFuc     KYTDFNS--------VKRGP-KRDLVGDLAKAVREAGL-----RFGVYYSGGLDWRFTTE          183
      . ::.       *   :   :  *  :  :     .  .  .  ::

BbAfcA      PLGAVALKSYEEELVKDP------------------RIAATMENAQKGEIMPNIPQMSA    339
BfFuc      PQGRTGRR-TGRPNPKGDMAHHHHHHSAALEVLFQGWKSYYQFMNNQLTELLTNYGPIGA
           200
TmarFuc     PIRYPEDLSYIRPNT----------------------YEYADYAYKQVMELVDLYLP-DV        220
      *         .                    *  *::     ..

BbAfcA      FWYAVRTAVINAASGRQTVDEALKDAQTNSSSNNNNNNNNNNLGENLYFQSVIASVEDGG
           399
BfFuc      IWFDG----------------------------------------------------W          206
TmarFuc     LWNDM----------------------------------------------------G        226
      :*

BbAfcA      DGDTSKDDWLWY--KQPASQTDATATAGGNYGNPDNNRWQQTTL-PF--------GNGKI     448
BfFuc      WDQDINPDFDWELPEQYALIHR--LQ----PACLVGNNHHQTPFA----GEDIQIFERDL         256
TmarFuc     WPEKGKEDLKYLFAYYYN------KH----PEGSVNDRW-GVPHWDFKTAEYHVNYPGDL     275
        : : * :                   .:.   .            .:

BbAfcA      GGTVWGEVSRERVTFNEETLWTGGPGSSTSYNGGNNETKGQNGATLRA----LNKQLANG
           504
BfFuc      PGENTAGLSGQSVSHLPLE-TCETMNGMWGYKIT--D---QNYKSTKTLIHYLVKAAGKD       310
TmarFuc     PGYKWEF--------------TRGIGLSFGYNRNEGP---EHMLSVEQLVYTLVDVVSKG 318
       *                .  .*:      ::  : .    * .  .:.

BbAfcA      -AETVNPGNLTGGENAAE-QGNYLNWGDIYLDYGFNDTTVTEYRRDLNLSKGKADVTFKH
           562
BfFuc      ANLLMNIGPQPDGELPEVAVQRLKEVGEWMSKYGETIY----------------------        348
TmarFuc     GNLLLNVGPKGDGTIPDLQKERLLGLGEWLRKYGDAIY----------------------       356
         :**   .*        .    *:   .**

BbAfcA      DGVTYTREYFASNPDNVMVARLTASKAGKLNFNVSMPTNTNYSKTGETTTVKGDTLTVKG
           622
BfFuc      ------------------------------------------------------------  348
TmarFuc     ------------------------------------------------------------              356


BbAfcA      ALGNNGLLYNSQIKVVLDNGEGTLSEGSDGASLKVSDAKAVTLYIAAATDYKQKYPSYRT
           682
BfFuc      ------------------------------------------------------------  348
TmarFuc     ------------------------------------------------------------              356


BbAfcA      GETAAEVNTRVAKVVQDAANKGYTAVKKAHIDDHSAIYDRVKIDLGQSGHSSDGAVATDA
           742
BfFuc      ------------------------------------------------------------  348
TmarFuc     ------------------------------------------------------------              356
```

```
BbAfcA     LLKAYQRGSATTAQKRELETLVYKYGRYLTIGSSRENSQLPSNLQGIWSVTAGDNAHGNT
           802
BfFuc      ------------------------------------------------GTRGGLVAP--H      358
TmarFuc    ------------------------------------------------GTSVWERCCAKT      368
                                                           ..    .

BbAfcA     PWGSDFHMNVNLQMNYWPTYSANMGELAEPLIEYVEGLVKPGRVTAKVYAGAETTNPETT
           862
BfFuc      DWGV---TTQ--------------------------------------------------      365
TmarFuc    EDGTEIRFTR--------------------------------------------------      378
            *    .

BbAfcA     PIGEGEGYMAHTENTAYGWTAPGQSFSWGWSPAAVPWILQNVYEAYEYSGDPALLDRVYA
           922
BfFuc      ------------------------------------------------------------      365
TmarFuc    ------------------------------------------------------------      378


BbAfcA     LLKEESHFYVNYMLHKAGSSSGDRLTTGVAYSPEQGPLGTDGNTYESSLVWQMLNDAIEA
           982
BfFuc      ------------------------------------------------------------      365
TmarFuc    ------------------------------------------------------------      378


BbAfcA     AKAKGDPDGLVGNTTDCSADNWAKNDSGNFTDANANRSWSCAKSLLKPIEVGDSGQIKEW
           1042
BfFuc      ------------------------------------------------------------      365
TmarFuc    ------------------------------------------------------------      378


BbAfcA     YFEGALGKKKDGSTISGYQADNQHRHMSHLLGLFPGDLITIDNSEYMDAAKTSLRYRCFK
           1102
BfFuc      --------------------KGNKLYVHILNLQDKALFLP--------------------      385
TmarFuc    --------------------KCNRIFVIFLGIPTGEKIV---------------------      397
                               : ::   :*.:    :

BbAfcA     GNVLQSNTGWAIGQRINSWARTGDGNTTYQLVELQLKNAMYANLFDYHAPFQIDGNFGNT
           1162
BfFuc      ------------------------------IVDKKVK-----------------------      392
TmarFuc    ------------------------------IEDLNLS-----------------------      404
                                         : : ::.

BbAfcA     SGVDEMLLQSNSTFTDTAGKKYVNYTNILPALPDAWAGGSVSGLVARGNFTVGTTWKNGK
           1222
BfFuc      ------------------------------------K--AVV------FA--------D      399
TmarFuc    ------------------------------------AGTVRH------FL--------T      413
                                                .      *

BbAfcA     ATEVRLTSNKGKQAAVKITAGGAQNYEVKNGDTAVNAKVVTNADGASLLVFDTTAGTTYT
           1282
BfFuc      KTPVRFTK-------------------NKEGIVLELAKVPTDVD---YVVELTID----- 432
TmarFuc    GERLSFKN-------------------VGKNLEITVPKKLLETDSITLVLEAVEE-----      449
            : :..                      :.     *  :.*   ::  .

BbAfcA     ITKKASGGSGGSHHHHHHHHGGSGGSWSHPQFEKGGGSGGGSGGSSAWSHPQFEK      1337
BfFuc      -------------------------------------------------------      432
TmarFuc    -------------------------------------------------------      449
```
